# Research on a Calculation Model of Ankle-Joint-Torque-Based sEMG

**DOI:** 10.3390/s24092906

**Published:** 2024-05-02

**Authors:** Xu Qiu, Haiming Zhao, Peng Xu, Jie Li

**Affiliations:** 1 College of Mechanical and Electrical Engineering, Central South University, Changsha 410083, China; qx021113@163.com (X.Q.); xp13125398052@163.com (P.X.); jieli_csu@163.com (J.L.); 2 State Key Laboratory of High Performance Complex Manufacturing, Central South University, Changsha 410083, China

**Keywords:** ankle joint torque, sEMG, Hill model, genetic algorithm

## Abstract

The purpose of this article is to establish a prediction model of joint movements and realize the prediction of joint movemenst, and the research results are of reference value for the development of the rehabilitation equipment. This will be carried out by analyzing the impact of surface electromyography (sEMG) on ankle movements and using the Hill model as a framework for calculating ankle joint torque. The table and scheme used in the experiments were based on physiological parameters obtained through the model. Data analysis was performed on ankle joint angle signal, movement signal, and sEMG data from nine subjects during dorsiflexion/flexion, varus, and internal/external rotation. The Hill model was employed to determine 16 physiological parameters which were optimized using a genetic algorithm. Three experiments were carried out to identify the optimal model to calculate torque and root mean square error. The optimized model precisely calculated torque and had a root mean square error of under 1.4 in comparison to the measured torque. Ankle movement models predict torque patterns with accuracy, thereby providing a solid theoretical basis for ankle rehabilitation control. The optimized model provides a theoretical foundation for precise ankle torque forecasts, thereby improving the efficacy of rehabilitation robots for the ankle.

## 1. Introduction

According to Feigin Valery L et al. [1], the global lifetime risk of stroke after the age of 25 years has increased from 22.8% in 1990 to 24.9% in 2016. From 2000 to 2050, the world’s elderly population will increase from 11% to 22%, with the absolute number increasing from 605 million to 2 billion. In China, there are already 200 million elderly people, and more than 50% of them suffer from physical disabilities [2]. In China, more than 50% of the elderly population suffers from physical disabilities, and ankle joint disorders account for a large proportion of the physical disabilities. In daily life, ankle sprains are one of the most common musculoskeletal injuries, accounting for 10–20% of all sports injuries [3]. The ankle joint plays an important role in maintaining balance and stabilizing gait, bears almost all the body weight, is subjected to large loads and impacts, and is highly susceptible to injury; so, a safe and reliable training device is needed for the injured ankle joint. In the process of ankle rehabilitation training, ankle joint movement is an important parameter of ankle rehabilitation booster control [1]. The ankle joint movements measured directly has a certain lag, while the prediction of joint torque through electromyography signals can realize the real-time tracking and measurement of ankle joint movement.

For the calculation of joint movements, Zoss A B et al. [4] calculated the joint movements based on human kinematics and dynamics modeling, and the calculated curves did not exactly match the measured curves. Chang Tongbo et al. [5] comparatively analyzed the biomechanical response of ankle cartilage and ligament during taijiquan knee-walking and walking, but did not take into account the influence of leg muscle groups on the ankle joint. Shao Q et al. [6,7,8,9,10] used electromyography and joint kinematics data as inputs to calculate joint movements by musculoskeletal modeling, and compared the output of the model with the results of inverse kinematics calculations or real measurements to validate the predictive ability of the method. Most of the physiological parameters in the model were from anatomy. The physiological parameters were not normalized, and the established model had the problem of individual variability. In addition to the calculation model of joint movements established under the physiological model, there are also mathematical methods applied to the calculation of joint movements; for example, nowadays, there are many studies on predicting joint movements by using mathematical methods combining the electromyographic signals of muscles, kinematic signals, and intelligent recognition algorithms [11,12,13].

To address the above problems, this paper establishes a myoelectric-signal-driven ankle movement calculation model based on the musculoskeletal model and uses a genetic algorithm to optimize the physiological parameters in the model, and the obtained optimized model is examined by the ankle rehabilitation experimental bench.

This paper makes innovations in the following sections:

- Analyze the data acquisition requirements and acquisition scheme, design the data acquisition circuit and fabricate the acquisition circuit board, and combine the software and hardware to form the robot data acquisition system.
- Based on the Hill model, the ankle joint movement calculation model is established, the experimental scheme is designed, the experimental data are collected, and the model parameters are optimized by using a genetic algorithm.

## 2. Materials and Methods

### 2.1. Calculation Model of Ankle Joint Movement

Computational modeling of ankle movements is important for designing myoelectrically controlled ankle rehabilitation mechanisms, studying functional electrical stimulation of atrophied muscles, and investigating how the nervous system controls limb movement [14]. The modeling of ankle joint movement is important for the design of ankle rehabilitation mechanisms. An ankle movement computational model developed in this paper takes kinematic and electromyographic signals from experimental subjects as inputs for estimating muscle forces associated with ankle movements. The model consists of two sub-models: the muscle activation model, and the musculoskeletal model.

#### 2.1.1. Muscle Activation Modeling

The magnitude of the EMG signal is controlled by neural commands; however, it is difficult to directly compare the absolute magnitude of two EMG signals because the magnitude of the EMG signal depends on the type of electrode, the position of the electrode with respect to the point of muscle movement, the amplifier gain, etc.; and in order to use the EMG in neuromusculoskeletal modeling, it needs to be transformed into a muscle activation, which has a time-varying value between 0 and 1.

The purpose of the muscle activation model is to synthesize the basic properties of muscles and tendons to study the equations of muscle activation dynamics [15]. The surface EMG signals of lower limb tibialis anterior, medial head of gastrocnemius, lateral head of gastrocnemius, and soleus were collected; the collected raw muscle EMG signals were processed by filtering and rectifying; the EMG data of maximal voluntary contraction were processed in the same way as described above; the EMG data of all the muscles in the experiments were divided by the maximum of the maximum voluntary contraction EMG data of the muscle, which means that EMG signals were normalized to obtain *e*(*t*), and the relationship between it and the neural activation *u*(*t*), and its relationship with the nerve activation *u*(*t*) is as follows:

(1) $$u\left(t\right)=\alpha e\left(t-d_{e}\right)-\beta_{1}u\left(t-1\right)-\beta_{2}u(t-2)$$

where $d_{e}$ denotes the electromechanical delay, which generally takes the value of 10–100 ms [16]. $\alpha ,\;\;\beta_{1},{\;\beta }_{2}$ are the neural activation coefficient.

The relationship between muscle activation and neural activation [17] is as follows:

(2) $$a\left(t\right)=\frac{e^{Au\left(t\right)-1}}{e^{A}-1}$$

where A∈(−3,0), is the nonlinear shape factor.

#### 2.1.2. Neuromusculoskeletal Geometric Modeling

**Figure 1:**
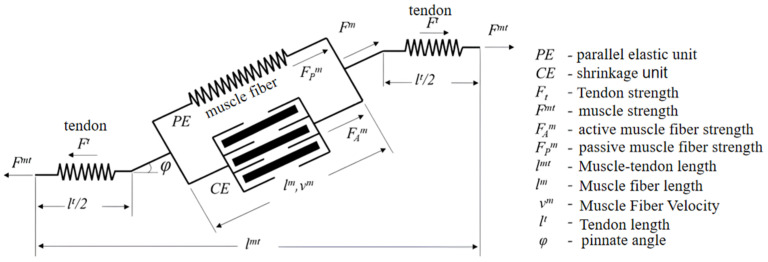
Schematic diagram of the muscle–tendon unit.

The muscle–tendon unit is shown in Figure 1, where the tendon is connected in series with the muscle fibers, which can be simplified to consist of contractile and parallel elastic units, and the contractile unit generates the active muscle fiber force ${F_{A}}^{m}$. The muscle fiber length is $l^{m}$ and the muscle fiber velocity is $v^{m}$. The active muscle fiber force generated by the contractile unit is a function of muscle fiber length and muscle fiber velocity, and the tendon force $F^{t}$ is a function of tendon length $l^{t}$, and the total muscle fiber force $F^{m}$ is a function of active muscle fiber force ${F_{A}}^{m}$ and passive muscle fiber force ${F_{P}}^{m}$. The sum of active and passive muscle fiber forces, and the muscle force generated by the whole muscle–tendon unit $F^{mt}$ is as follows:

(3) $$F^{mt}=F^{t}={{(F}_{A}}^{m}+{F_{P}}^{m})\cos\varphi$$

The time-varying muscle fiber fractional force generated by the contractile unit ${F_{A}}^{m}\left(t\right)$ is as follows:

(4) $${F_{A}}^{m}\left(t\right)=f_{A}(v)f_{A}(l)a(t){F_{0}}^{m}$$

where ${F_{A}}^{m}\left(t\right)$ is the muscle fiber force generated by CE, $f_{A}(v)$ is the velocity-dependent active muscle fiber force, $f_{A}(l)$ is the length-related active muscle fiber force, a(t) is time-varying muscle activation, ${F_{0}}^{m}$ is the maximum muscle fiber force, l is the muscle length, and v is the muscle contraction velocity.

The time-varying muscle fiber component force generated by parallel elastic units ${F_{P}}^{m}\left(t\right)$ is as follows:

(5) $${F_{P}}^{m}\left(t\right)=f_{P}(l){F_{0}}^{m}$$

where $f_{P}(l)$ is the normalized length-dependent passive muscle fiber force.

(1) Relationship between muscle force and muscle fiber length

Muscle fibers can be thought of as consisting of active and passive units, with the active unit generating force when activated, similar to a motor, and the passive unit generating resistance when stretched beyond a certain length, similar to a rubber band; human muscle strength reaches its maximum when the length of the muscle knob is 2.64 μm [18]. When a muscle fiber has a knob length of 2.64 μm, human muscle strength reaches its maximum value, and when the knob reaches this value, it is considered to be at the optimal muscle fiber length ${l_{0}}^{m}$. 

Normalized muscle length is as follows:

(6) $$l=\frac{l^{m}}{{l_{0}}^{m}}$$

Time-varying optimal muscle length ${l_{0}}^{m}\left(t\right)$ is related to muscle activation in the following way:

(7) $${l_{0}}^{m}\left(t\right)={l_{0}}^{m}(\lambda \left(1-a\left(t\right)\right)+1)$$

where λ is the percentage change in muscle fiber length.

Based on the literature [19], normalized length-related active muscle fiber force is as follows:

(8) $$f_{A}\left(l\right)\left\{\begin{array}{c} q_{0}+q_{1}l+q_{2}l^{2}\;\;0.5\leq l\leq 1.5\\ 0\;\;\;\;\;\;\;\;\;\;\;\;\;\;\;\;\;\;else \end{array}\right.$$

$q_{0}=-2.06$. $q_{1}=6.16$. $q_{2}=-3.13$

Normalized length-related passive muscle fiber force [20] $f_{P}\left(l\right)$ is as follows:

(9) $$f_{P}\left(l\right)=\frac{e^{10(l-1)}}{e^{5}}=e^{10l-15}$$

(2) Relationship between muscle force and rate of muscle contraction

Muscle fiber force is related to the rate of contraction of a muscle in addition to the length of the muscle fiber [15]. The Hill model was first used to detect the heat associated with muscle contraction, and Hill found that when a muscle contracts by a length of *x*, it emits heat of contraction *H* [21]:

(10) H=ax

where *a* is a thermal constant related to the cross-sectional area of the muscle.

Hill considered that from the point of view of conservation of energy, the total energy *E* produced by the muscle is the energy sum of the heat dissipation and the work carried out by the muscle fiber force:

(11) $$E=F^{m}x+H=(F^{m}+a)x$$

The above equation differentiates the time t to obtain the energy release rate as follows:

(12) $$\frac{(F^{m}+a)dx}{dt}=(F^{m}+a)v^{m}$$

Hill suggested experimentally that $\left(F^{m}+a\right)$ is a linear function of muscle strength $F^{m}$ of a linear function of the muscle force:

(13) $$\left(F^{m}+a\right)v^{m}=b({F_{0}}^{m}-F^{m})$$

where *b* is the absolute rate of energy release; a constant, as in the above equation, can be obtained by organizing the following formula:

(14) $$F^{m}=\frac{b{F_{0}}^{m}-av^{m}}{b+v^{m}}$$

Equation (14) considers only muscle contraction, taking into account the muscle force during muscle diastole, muscle force f-velocity v. The relationship can be simplified as in [22] the following equation:

(15) $$f(v)=\left\{\begin{array}{c} \begin{array}{cc} \frac{0.3\left(\frac{v^{m}}{v_{0}^{m}}+1\right)}{-\frac{v^{m}}{v_{0}^{m}}+0.3} & \frac{v^{m}}{v_{0}^{m}}<0 \end{array}\\ \begin{array}{cc} \frac{2.34\frac{v^{m}}{v_{0}^{m}}+0.039}{1.3\frac{v^{m}}{v_{0}^{m}}+0.039} & \frac{v^{m}}{v_{0}^{m}}\geq 0 \end{array} \end{array}\right.$$

(3) Tendon models

**Figure 2:**
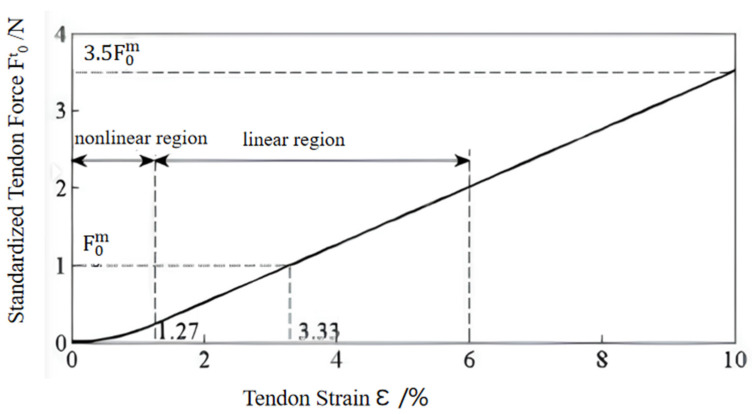
Tendon force–strain relationship [15].

According to Hill’s model, muscle fibers are connected in series with the tendon and the tendon strain is defined as follows:

(16) $$\varepsilon^{t}=\frac{l^{t}-{l_{s}}^{t}}{{l_{s}}^{t}}$$

where ${l_{s}}^{t}$ is the relaxation length of the tendon, $l^{t}$ is the time-varying tendon length, and the tendon strain $\varepsilon^{t}$ cannot be negative. When $l^{t}\leq {l_{s}}^{t}$ the tendon is not subjected to any load, the tendon force is zero; when $l^{t}>{l_{s}}^{t}$ time, the tendon force is related to its stretched length. Zajac found that the relationship between tendon force and tendon strain is shown in Figure 2, and the functional relationship can be defined as follows:

(17) $$F^{t}={F_{0}}^{m}\times \left\{\begin{array}{c} 0\;\;\;\;\;\;\;\;\;\;\;\;\;\;\;\varepsilon \leq 0\\ 1480.3\varepsilon^{2}\;\;0<\varepsilon <0.0127\\ 37.5\varepsilon -0.24\;\;\varepsilon \geq 0.0127 \end{array}\right.$$

(4) Pinnae and muscle tendon lengths

The pinnation angle is the angle between the tendon and the muscle fibers, and because the tendon is in series with the muscle fibers, the tendon force can be defined as follows:

(18) $$F^{t}=F^{m}\cos\varphi$$

where φ is the pinnation angle. When φ is small, its effect on the muscle–tendon unit’s force is limited, and when φ larger, its effect on force can be large. It has been shown that the pinnation angle of a given muscle is not constant, and it changes with joint angle and muscle activation [23]. The results of this study are summarized in the following table. Scott et al. [24] developed a simple model which quickly calculates the pinnation angle:

(19) $$\varphi \left(t\right)=\sin^{-1}\frac{{l_{0}}^{m}\sin\varphi_{0}}{l^{m}(t)}$$

where $l^{m}(t)$ is the time-varying muscle fiber length, and $\varphi_{0}$ is the optimal muscle fiber length ${l_{0}}^{m}$ of the pinnation angle at a specific time.

From Section 2.1.1 and Section 2.1.2, it is clear that tendon length is important in solving for muscle fiber force following the Hill muscle model:

(20) $$l^{t}=l^{mt}-l^{m}\cos\varphi$$

(5) Muscles seek to solve

The relationship between muscle fiber force and muscle contraction according to Equations (3)–(5) is solved as follows:

(21) $$f\left(v\right)=\frac{F^{t}-f_{P}\left(l\right){F_{0}}^{m}\cos\varphi }{f_{A}(l)a(t){F_{0}}^{m}\cos\varphi }$$

(6) Solving for joint movements

Joint movements are the sum of the muscle tendon forces multiplied by their respective force arms; the geometry of the musculoskeletal bones determines the muscle force arms, which are not constant values, but are time-varying with the joint angle:

(22) $$M\left(\theta ,t\right)=\sum_{i=1}^{m}\left(r_{i}(\theta )\cdot {F_{i}}^{mt}(\theta ,t)\right)$$

where $M\left(\theta ,t\right)$ is the joint movement, ${F_{i}}^{mt}(\theta ,t)$ is the muscle force, $r_{i}(\theta )$ is the muscle force arm, *t* is the sampling movement, the θ is the joint angle, and *i* is the muscle identification.

The force arm solution equation is as follows:

(23) $$r\left(\theta \right)=\frac{\partial l^{mt}(\theta )}{\partial \theta }$$

#### 2.1.3. Parametric Analysis

Based on [25] studies and model tests, in the muscle activation model, the electromechanical delay $d_{e}$ is taken as 40 ms, α=0.9486. The $\beta_{1}=-0.052$, $\beta_{2}=0.000627$, and A=−1.5, and in the Hill model, the λ is set at 15%. ${F_{0}}^{m}$, ${l_{0}}^{m}$, ${l_{s}}^{t}$, ${v_{0}}^{m},\;\varphi_{0}$ are constants related to the human body, and for different muscles, the initial values are shown in Table 1, while $l^{mt}$, v, r(θ), $l^{m}$ are time-varying variables, and according to [26,27] studies, the time-varying muscle–tendon length can be approximated by a fitting polynomial of the joint angle, as shown in Equation (24):

(24) $$l^{mt}\left(\theta \right)=\mu_{0}+\mu_{1}\theta \left(t\right)+\mu_{2}{\theta \left(t\right)}^{2}+\mu_{3}{\theta \left(t\right)}^{3}$$

In the equation, $\mu_{0}$, $\mu_{1}$, $\mu_{2}$, and $\mu_{3}$ are constant coefficients of the polynomial. Thus, the muscle force arm r(θ) can be expressed as follows:

(25) $$r\left(\theta \right)=\mu_{1}+{2\mu }_{2}\theta (t)+3\mu_{3}{\theta (t)}^{2}$$

From the given body-related constants, the muscle force can be found from Equation (15), which, in turn, can be combined with Equation (21) to find the muscle fiber velocity v, and muscle fiber length $l^{m}$ can be obtained by utilizing the Lunger–Kutta algorithm for the v forward integration [25].

#### 2.1.4. Parameter Optimization

Analyzing the literature [25], it was found that most of the model parameter values were taken from the anatomical model, and there were some differences in the specific values, which could not respond well to the variability between individuals; in order to adjust the model parameters, this paper assumes that the joint movements measured by the experimental device and the movements obtained from the joint movement computation model are equal; thus, Equation (26) is used to express the relationship between them:

(26) $$min\sum_{i=1}^{m}{\left(M_{i}-\sum_{j=1}^{n}F_{ij}\times r_{ij}\right)}^{2}$$

where *M* denotes the measured joint movement, *F* denotes muscle force, *r* denotes muscle force arm, *i* is different muscle labeling, and *j* is the sampling point.

In this paper, the initial value of 16 parameters is taken as the benchmark, and the value interval of the parameters is set, as shown in Table 1; the value interval of the parameters is summarized according to the distribution range of the data within previous studies, and the original value within the literature is taken because the speed has a small impact on the final calculation results. The genetic algorithm is applied to find the optimal in the parameter interval using Equation (26) as the fitness function, and the binary coding is adopted in the optimization process, setting the initial population number is 100. The maximum number of evolutionary generations is 150, the selection operation adopts a roulette, the probability of single-point crossover is 0.6, and the probability of basic positional variation is 0.1 to obtain the final optimal parameter set.

### 2.2. Experimental Program

#### 2.2.1. Lab Bench Design

(1) Structural design

**Figure 3:**
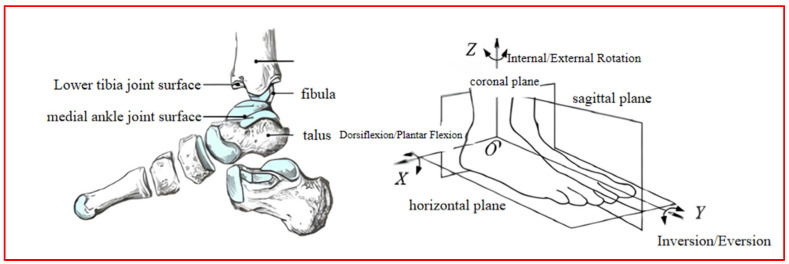
Ankle joint anatomy.

The ankle joint is one of the most important weight-bearing parts of the body, mainly composed of the tibia, fibula, and talus, and its movements mainly include internal/external rotation in the horizontal plane, internal/external rotation in the coronal plane, and dorsiflexion/plantar flexion in the sagittal plane [30]. The structure and movement type of the ankle joint is shown in Figure 3. 

**Figure 4:**
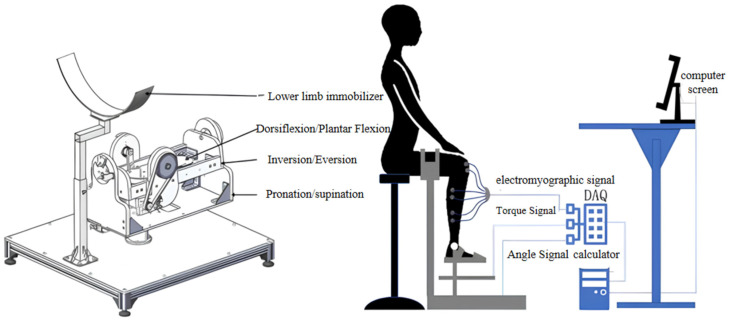
Ankle rehabilitation laboratory.

According to the demand of ankle rehabilitation movement, the ankle rehabilitation device was designed, including a mechanical structure, signal acquisition system and control system. Its mechanical structure model is shown in Figure 4, which mainly includes four parts: the internal and external rotation mechanism, the inversion and eversion rotation mechanism, the dorsiflexion and toe flexion mechanism, and a lower limb fixation frame. The three-degree-of-freedom rotary motion is driven by DC servomotor.

The lower limb fixation frame is adjustable in the height direction to adapt to different height objects. In order to ensure safety, according to the physiological structure characteristics of the ankle joint, the electrical limit and mechanical limit are designed in the direction of three degrees of freedom, and the redundant design of the protection device can improve the safety of rehabilitation training.

**Figure 5:**
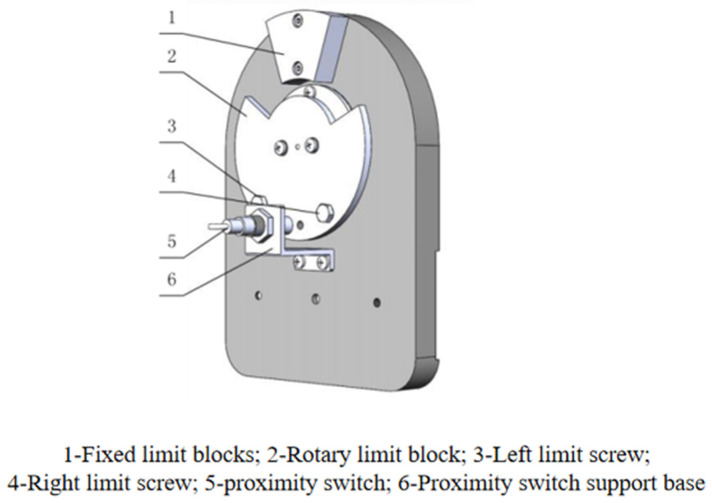
Limit structure of the dorsiflexion/plantar flexion mechanism.

To ensure safety, according to the physiological structure of the ankle joint, mechanical limits and electrical limits are designed in the direction of three degrees of freedom. The protection redundancy can not only ensure the patient’s safety but also protect the machine from damage. Figure 5 shows the limit structure of the dorsiflexion/plantar flexion mechanism; the mechanical limit will be triggered only when the electrical limit fails. The rest of the limiting mechanism in the direction of the two degrees of freedom is similar to this mechanism.

The mechanical limiting mechanism includes fixed limiting block 1 and rotary limiting block 2. The fixed limiting block is fixedly connected to the bearing seat, while the rotary limiting block is fixedly connected to the dorsiflexion/toe flexion rotary axis so that the dorsiflexion/toe flexion axis is restricted to rotate within a certain range. To realize the mechanical limiting, the angle of the mechanical limiting is larger than the angle of the demand for rehabilitation by 2°, and it will not trigger mechanical limiting in normal operation, although will trigger the mechanical limiting in the case of malfunction of the operation and will not cause injury to the ankle joint. The electrical limit mechanism includes left limit screw 3, right limit screw 4, proximity switch 5, and proximity switch support seat 6. The limit screw is fixedly connected to the rotary limit block. When the proximity switch senses the limit screw during the movement of the mechanism, the proximity switch realizes a trip, and the controller will change the position accordingly. When the proximity switch senses the limit screw during the movement of the mechanism, the proximity switch realizes jumping, and the controller controls the mechanism to complete the commutation movement according to the jump signal.

(2) Signal Acquisition System Design

In order to realize the intelligent control of the ankle rehabilitation device and the feedback of the rehabilitation situation, a signal acquisition system was designed, and the schematic diagram of the experimental device is shown in Figure 4. This system can acquire the electromyographic signals of the ankle action muscle groups, the joint movement signals, and the joint angle signals during the rehabilitation process.

**Figure 6:**
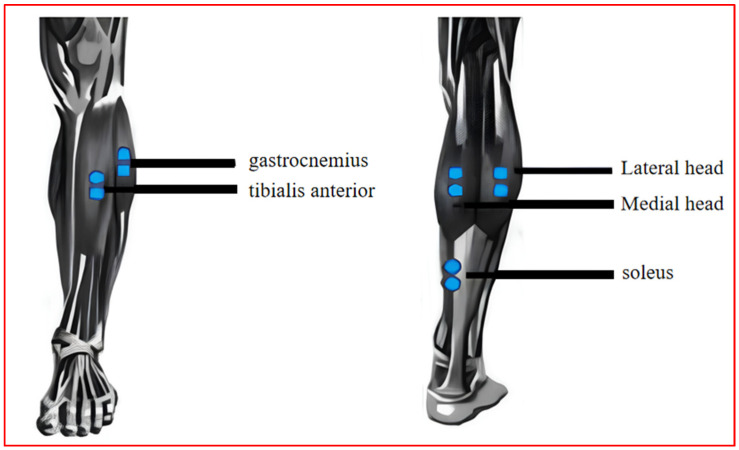
sEMG sensor layout scheme.

In order to ensure the safety and simplicity of the EMG acquisition process, this paper uses patch electrodes to acquire surface EMG signals [31]. The surface EMG signal sensor includes a bipolar patch electrode and a reference patch electrode. The surface EMG signal sensor includes bipolar patch electrodes and reference patch electrodes. The bipolar electrodes are arranged at the two ends of the muscle; the larger the muscle, the farther the electrode spacing. The preferred distance is 20 mm [32], and the reference electrode is arranged on the inactive (tendon or bone) tissue. The location of the surface EMG sensor arrangement on the leg is shown in Figure 6.

Based on the characteristics of myoelectric signals, the surface muscle electric sensor (EDK0005) was utilized to collect the lower limb muscle electric signals.

The sensor adopts an AD8221 chip, which can convert the differential input signal into a single-ended signal output, and it will collect the original electromyographic signal for adjustable amplification and the signal output after filtering and rectification. The power supply voltage of the module is DC 3.5~18 V, the electrode lead port is a 3.5mm headphone hole, the working temperature is −20~+60 °C, the module size is 2.54 cm × 2.54 cm × 2.54 cm, which is small in area, and the electrical specifications of the module are shown in Table 2. 

**Figure 7:**
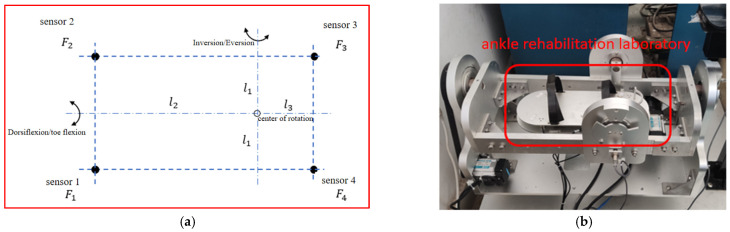
Tension and pressure sensor layout scheme. (**a**) Schematic diagram of sensor arrangement. (**b**) Real picture of experimental setup.

The joint movement signals were collected jointly by the movement sensor and the pull-pressure sensor. The internal and external rotation movement signals were directly collected by the movement sensor, and the dorsiflexion/toe flexion and inversion/eversion movement signals were indirectly collected by the pull-pressure sensor, which is arranged at the sole of the foot, and the arrangement scheme is shown in Figure 7.

Torque value $T_{i}$ is the signal of the tensile transducer $F_{i}$ and the corresponding force arm $l_{i}$ of the corresponding force arm, as follows:

(27) $$T_{i}=F_{i}\times l_{i}$$

Among them: $l_{1}=50\;mm,\;l_{2}=200\;mm$,$\;l_{3}=40\;mm$.

The joint angle signal is obtained by acquiring the DC servo motor encoder signal.

#### 2.2.2. Implementation Program

The subjects of this study were 9 males, the age of the subjects was 23 ± 2 years old, the height was 170 ± 10 cm, and the weight was 70 ± 10 Kg. The subjects had no history of lower limb injury, and before the experiment was carried out, sufficient rest was given to ensure that the subjects had no lower limb fatigue, and informed consent was obtained from the subjects for this experiment.

**Figure 8:**
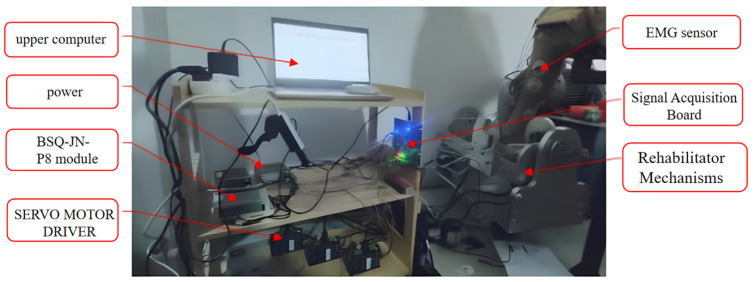
Image of experiment site.

The experimental subject’s upper body is straight, sitting on the chair in front of the test bench. The thigh of the right leg of the subject is placed on the thigh support bracket of the test bench, the thigh and calf are kept in 90° bending, the soles of the feet are in contact with the rehabilitation device, and the soles of the feet and the calves are also at 90°; the soles of the feet and the thighs are secured with a magic bandage to prevent the position of the experiment from being changed during the experiment. Four pairs of electrodes were affixed to the tibialis anterior, medial head of gastrocnemius, lateral head of gastrocnemius and soleus muscles, and when the electrodes were affixed, the skin tissue of the experimental subjects was disinfected and shaved, and the connecting lines between the electrode pieces were parallel to the long axis of the muscle fibers. During the experiment, the experimental subject kept the body posture unchanged, and only the ankle joint moved, and the picture of the experimental bench site is shown in Figure 8.

Each group of experiments includes three movements, dorsiflexion/toe flexion, internal rotation and internal/external rotation. The human body’s own weight becomes a load during walking, and in order to simulate its own load, the experimental device applies a certain amount of damping force. The acquisition of the object’s right lower limb electromyographic signals, torque signals, and joint angle signals is carried out. According to the physiological characteristics of ankle joint movement, the movement angles in each direction are as follows: 30° dorsiflexion, 45° toe flexion, 15° internal rotation, 35° internal rotation, and 25° external rotation. Each group of experiments is conducted three times, with an interval of 3 min each time, so as to allow the experimental subjects to recover their physical strength, and to prevent experiments from being carried out under the intensity of fatigue.

## 3. Results

**Figure 9:**
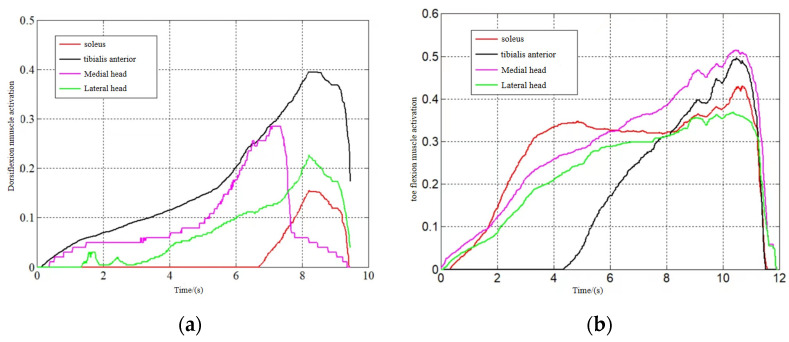
Dorsiflexion/toe flexion muscle activation. (**a**) Dorsiflexor activation. (**b**) Activation of toe flexor muscles.

In order to analyze the contribution of the four muscles to ankle motion, the muscle activation of the four muscles during the experiment was analyzed. As shown in Figure 9, in dorsiflexion, it was the tibialis anterior muscle that played a major role, followed by the gastrocnemius, with the flounder contributing less to this. In toe flexion, it was the medial and lateral heads of the gastrocnemius and the piriformis that played a major role in the early phase, with the tibialis anterior playing a role in the later phase when the angle was greater.

**Figure 10:**
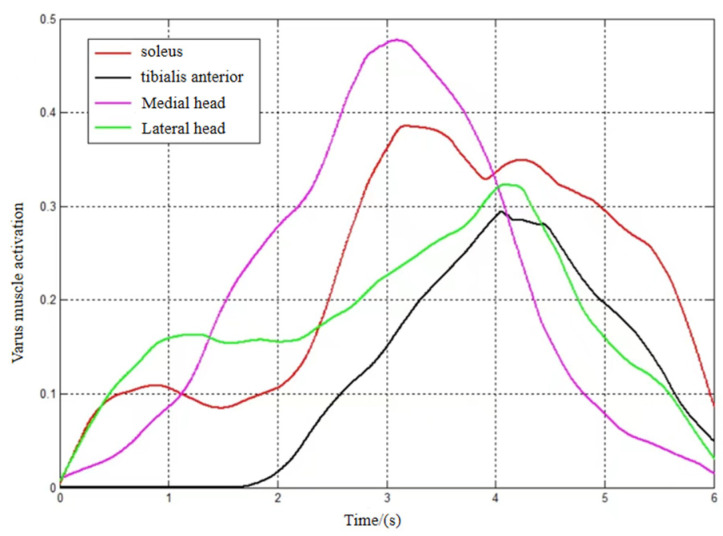
Varus muscle activation.

As shown in Figure 10, during inversion, the medial and lateral gastrocnemius and flounder muscles played a major role in the early stages, and the tibialis anterior muscle started to play a role in the later stages when the inversion angle was larger.

**Figure 11:**
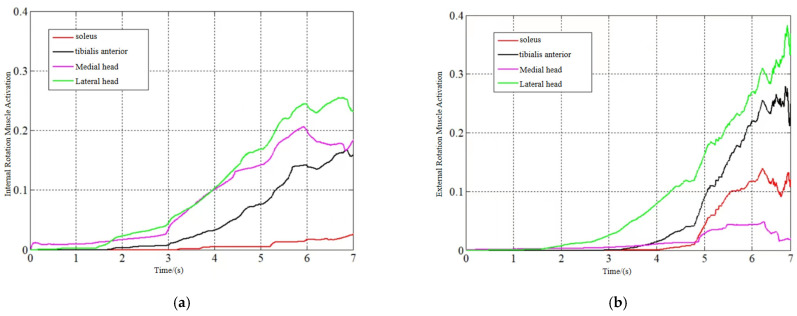
Internal/external rotation muscle activation. (**a**) Activation of endomorphic muscles. (**b**) External rotation muscle activation.

As shown in Figure 11, the flounder muscle contributed very little to the joint movement in internal rotation, and the medial head of the gastrocnemius contributed very little to the movement in external rotation.

Among the above movements in the three degrees of freedom directions, the dorsiflexion/toe flexion movement, the combined participation of the four muscles is the highest; so, if dorsiflexion/toe flexion is chosen as the main rehabilitation movement of the ankle joint, it can realize efficient rehabilitation. Muscle activation can respond to the magnitude of autonomous movements during ankle joint movements, and electromyography can be applied to the controller’s design.

The analysis of muscle activation has a certain reference value for ankle rehabilitation program development and diagnosis. It can be used to select the rehabilitation program according to the patient’s muscle damage to ensure the rehabilitation effect of the target muscle, and can also be used to determine the damaged muscle according to the difficulty of the patient’s ankle joint to perform the rehabilitation movement.

The optimization of model physiological parameters using experimental data from six subjects yielded an optimized parameter set for the ankle movement calculation model, with specific values shown in Table 3.

An optimization model was used to predict the ankle joint movements for each movement in three subjects, and the root mean square error between the predicted and measured movements was calculated.

**Figure 12:**
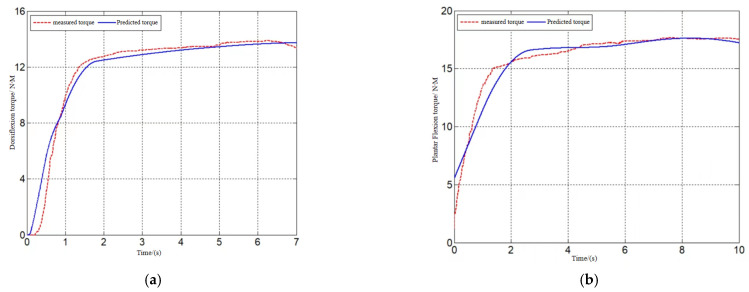
Dorsiflexion/toe flexion torque prediction. (**a**) Prediction of dorsiflexion movement. (**b**) Prediction of toe flexion movement.

**Figure 13:**
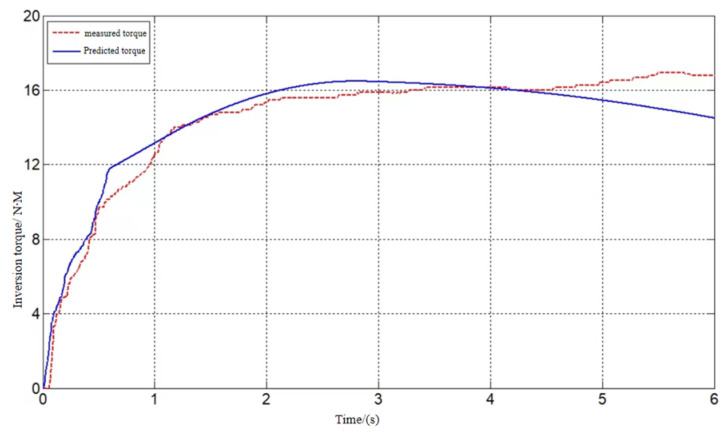
Inversion torque prediction.

**Figure 14:**
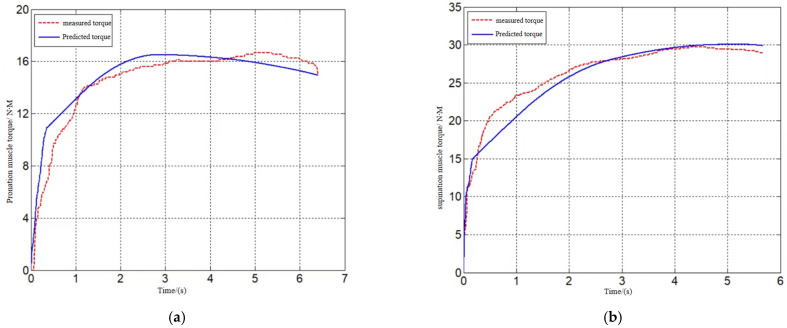
Internal/external rotation torque prediction. (**a**) Prediction of internal rotation movement. (**b**) Prediction of external rotation movement.

Figure 12, Figure 13 and Figure 14 show the model-predicted movement versus measured movement curves for 1 of the subjects. The poor model prediction at the onset of the movement was due to the deviation in the initial length assignment of the muscle fibers, and the initial length of the muscle fibers can be used as an optimization parameter to obtain a more accurate prediction model in subsequent studies.

## 4. Discussion

**Figure 15:**
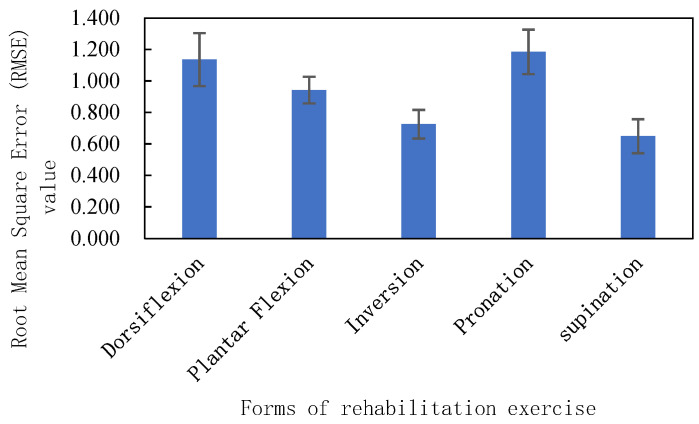
Root mean squared error of measured torque and predicted torque.

The root mean squared errors of measured and predicted movements in each motion are statistically presented in Figure 15. The root mean squared error characterizes the degree of the curve fit between the predicted and measured movements, and can be used to judge the accuracy of the prediction results. The root mean squared errors in the dorsiflexion/toe flexion directions were 1.136 ± 0.168 and 0.942 ± 0.085, respectively; and in endotropic directions, these were 0.725 ± 0.0.091; The root mean squared errors in the direction of internal/external rotation were 1.185 ± 0.141 and 0.649 ± 0.108, respectively.

According to the experimental results, it can be seen that the predicted movements of the model and the measured movements in each movement of the ankle joint have the same trend, and the corresponding time movement values are not much different; so, the accuracy of the ankle joint movement calculation model is better, it meets the requirements of the ankle joint rehabilitation-device-assisted control, and it can be used to obtain a more accurate ankle joint movement calculation model that can adapt to the differences of the individuals by collecting more sample data of different age groups, different physical states and different genders. 

## 5. Conclusions

Based on Hill’s model, the relationship between surface muscle electrical signals and ankle joint movements was investigated, the ankle joint movement prediction model was established, the contribution of each muscle in different rehabilitation maneuvers was studied, and the following conclusions were obtained:

- Physiological parameters that have a greater effect on muscle force were identified;
- A model for calculating ankle joint movements based on surface muscle electrical signals was developed.

In this paper, the physiological parameters in Hill’s model were optimized using genetic algorithm from physiological parameters, the prediction accuracy of the ankle joint movement calculation model based on the surface muscle electrical signals established was better, and the torque that the patient’s ankle joint can produce can be predicted according to the collected surface muscle electrical signals in control according to the difference between predicted torque and the actual required torque, which can provide a quantitative way to realize the intelligent assisted force control. reference. At the same time, the model can be used to predict the joint movements of other joints, such as knee and hip, by combining appropriate physiological and anatomical data.

The ankle joint movement calculation model based on surface muscle electrical signals was established, which lays the foundation for the intelligent control of an ankle rehabilitation robot. The movement generated by the ankle joint can be predicted based on the collected surface electromyographic signals. For patients with lower limb disorders, the movement generated may not be enough to support them to complete the corresponding movements, and the control system can provide them with assistance based on the difference between the predicted movement and the actual required movement to realize the assistance control.

The shortcoming of this study is that the experimental sample lacks diversity. The next step of the experiment will consider sample data from different age groups, different physical states, and different genders to obtain an ankle movement calculation model that can adapt to individual variability and be more accurate.

## Figures and Tables

**Table 1:** Initial values of parameters in the neuromusculoskeletal model [28,29].

Parameters	Tibialis Anterior Muscle (Anatomy)	Flounder Muscle	Medial Head of Gastrocnemius	Lateral Head of the Gastrocnemius Muscle	Range of Values
Maximum muscle force ${F_{0}}^{m}/N$	1270	2830	1115	490	±50%
Optimal muscle fiber length ${l_{0}}^{m}/m$	0.031	0.030	0.045	0.064	±50%
Tendon relaxation length ${l_{s}}^{t}/m$	0.31	0.268	0.408	0.385	±15%
Feather angle at optimal muscle fiber length $\varphi_{0}/{}^{\circ}$	12	25	17	8	±50%

**Table 2:** Electrical parameter table for muscle electric sensors.

Parameters	Minimum Value	Regular Value	Maximum Values
Supply Voltage (Vs)	±3 V	±5 V	±18 V
Gain (207 × (x/1 kΩ))	0.01 Ω (0.002×)	50 kΩ (10,350×)	100 kΩ (20,700×)
output voltage	0 V	--	+Vs
Differential Input Voltage	0 mV	2–5 mV	+Vs/Gain

**Table 3:** The optimized parameter value is obtained by using genetic algorithm.

Parameters	Tibialis Anterior Muscle	Flounder Muscle	Medial Head of Gastrocnemius	Lateral Head of the Gastrocnemius Muscle
$\mathrm{Maximum}\;\mathrm{muscle}\;\mathrm{force}\;{F_{0}}^{m}/N$	1247.985	4070.933	1422.002	632.503
$\mathrm{Optimal}\;\mathrm{muscle}\;\mathrm{fiber}\;\mathrm{length}\;{l_{0}}^{m}/m$	0.027	0.032	0.050	0.059
$\mathrm{Tendon}\;\mathrm{relaxation}\;\mathrm{length}\;{l_{s}}^{t}/m$	0.314	0.309	0.326	0.422
$\mathrm{Feather}\;\mathrm{angle}\;\mathrm{at}\;\mathrm{optimal}\;\mathrm{muscle}\;\mathrm{fiber}\;\mathrm{length}\;\varphi_{0}/{}^{\circ}$	12.010	25.702	14.302	7.307

## Data Availability

The data presented in this study are available upon request from the first author.
